# Lipopolysaccharide Induces Human Pulmonary Micro-Vascular Endothelial Apoptosis via the YAP Signaling Pathway

**DOI:** 10.3389/fcimb.2016.00133

**Published:** 2016-10-19

**Authors:** Lei Yi, Xiaoqin Huang, Feng Guo, Zengding Zhou, Mengling Chang, Jiajun Tang, Jingning Huan

**Affiliations:** ^1^Department of Orthopedics, Shanghai Fengxian Central Hospital, Branch of The Sixth People's Hospital Affiliated to Shanghai Jiao Tong UniversityShanghai, China; ^2^Department of Burn and Plastic Surgery, Ruijin Hospital, School of Medicine, Shanghai Jiao Tong UniversityShanghai, China

**Keywords:** lipopolysaccharide, endothelial cells, Yes-associated protein, P73, apoptosis

## Abstract

Gram-negative bacterial lipopolysaccharide (LPS) induces a pathologic increase in lung vascular leakage under septic conditions. LPS-induced human pulmonary micro-vascular endothelial cell (HPMEC) apoptosis launches and aggravates micro-vascular hyper-permeability and acute lung injury (ALI). Previous studies show that the activation of intrinsic apoptotic pathway is vital for LPS-induced EC apoptosis. Yes-associated protein (YAP) has been reported to positively regulate intrinsic apoptotic pathway in tumor cells apoptosis. However, the potential role of YAP protein in LPS-induced HPMEC apoptosis has not been determined. In this study, we found that LPS-induced activation and nuclear accumulation of YAP accelerated HPMECs apoptosis. LPS-induced YAP translocation from cytoplasm to nucleus by the increased phosphorylation on Y357 resulted in the interaction between YAP and transcription factor P73. Furthermore, inhibition of YAP by small interfering RNA (siRNA) not only suppressed the LPS-induced HPMEC apoptosis but also regulated P73-mediated up-regulation of BAX and down-regulation of BCL-2. Taken together, our results demonstrated that activation of the YAP/P73/(BAX and BCL-2)/caspase-3 signaling pathway played a critical role in LPS-induced HPMEC apoptosis. Inhibition of the YAP might be a potential therapeutic strategy for lung injury under sepsis.

## Introduction

Sepsis is a serious consequence of Gram-negative bacterial infection in critically ill patients (Wendel et al., 2007). Under septic condition, the most frequently affected tissue is the endothelium lining the intimal surface of the vasculature (Cook-Mills and Deem, 2005). Lipopolysaccharide (LPS), as a major component of the outer membrane of Gram-negative bacteria, induces a multitude of endothelial dysfunctions, such as the coagulation activation and vascular barrier disruption (Zhou et al., 2013; Yi et al., 2016). Furthermore, LPS-induced endothelial cells (ECs) apoptosis and detachment from the vessel basement membrane deteriorates LPS-induced micro-vascular injury and further induces dysfunction in multiple organs (Hotchkiss et al., 2002). Recent studies have showed that the sepsis-induced acute lung injury (ALI) is characterized by apoptosis of the lung micro-vascular endothelial cells (Gill et al., 2014, 2015). However, the signaling transduction pathways implicated in LPS-induced human pulmonary micro-vascular endothelial cell (HPMEC) apoptosis remains unclear. In this study, we aim to define the molecular mechanism by which LPS induces apoptosis of HPMECs *in vitro*.

Yes-associated protein (YAP) is a key effector of the Hippo signaling pathway and is primarily inactivated by large tumor suppressor gene (LATS) in the cytoplasm via serine phosphorylation (Yu and Guan, 2013; Guo and Teng, 2015). YAP protein is also a crucial transcriptional co-activator and is involved in the regulation of cellular proliferation, differentiation, transformation and apoptosis (Wang et al., 2014; Hansen et al., 2015). When activated, YAP translocates from the cytoplasm to the nucleus of cells and binds to downstream transcription factors to regulate different biological functions (Strano et al., 2001; Vassilev et al., 2001; Komuro et al., 2003). Previous studies have reported that YAP is extensively activated and mediates tumor cell apoptosis in response to DNA damage (Zagurovskaya et al., 2009). However, whether YAP is implicated in LPS-induced HPMEC apoptosis has not been investigated.

To date, various transcription factors have been reported to be the YAP target proteins, such as TEAD, P73, Egr-1, Runx2, and ErbB-4 (Strano et al., 2001; Vassilev et al., 2001; Komuro et al., 2003; Zagurovskaya et al., 2009). P73 displays higher affinities for YAP and are positively regulated by YAP in tumor cell apoptosis (Strano et al., 2005). The phosphorylation of YAP on tyrosine 357 (T357) results in stabilization of YAP protein, which promotes interaction between YAP and P73 and further co-activates P73 downstream target genes (Levy et al., 2008). However, the interaction between YAP and P73 during LPS-induced HPMEC apoptosis has not been investigated.

The BCL-2 family is characterized by the BCL-2 homology domain, which mediates cellular apoptosis by regulating mitochondrial membrane permeability (Youle and Strasser, 2008). P73-mediated regulation of BCL-2 family proteins involves mechanisms related to many cells apoptosis (Tiwary et al., 2011). Therefore, we hypothesized that YAP was involved in LPS-induced HPMEC apoptosis via the activation of P73 and the subsequent regulation of BCL-2 family proteins.

In the present study, we found that YAP was activated during LPS-induced HPMEC apoptosis via the phosphorylation on Y357, which accumulated in the nucleus and bound transcription factor P73. Moreover, inhibition of YAP not only prevented EC apoptosis induced by LPS but also blocked the activation of P73 in LPS-stimulated HPMEs. Our results first reported the core effects of YAP/P73 signaling in LPS-induced HPMEC apoptosis, which might play an important pathogenic role in sepsis-induced lung injury.

## Materials and methods

### Cell culture and reagents

Primary HPMECs were obtained from ScienCell Research Laboratories and were maintained in ScienCell Endothelial Cell Medium in a humidified incubator at 37°C with 5% CO_2_. LPS (from *Escherichia coli* 055:B5) and cycloheximide (CHX) were purchased from Sigma-Aldrich (St Louis, MO). The anti-YAP rabbit monoclonal antibody and the anti-YAP (phospho-Y357) rabbit polyclonal antibody were obtained from ABCAM. The anti-BAX rabbit mAb, anti-BCL-2 rabbit mAb, anti-P73 mouse mAb, anti-cleaved caspase-3 rabbit mAb, anti-cleaved-PARP rabbit mAb and anti-rabbit immunoglobulin-G-HRP-linked antibody were purchased from Cell Signaling Technologies (Danvers, Mass). The anti-mouse immunoglobulin-G-HRP-linked antibody, anti-pro-caspase-3 mouse mAb, anti-PARP mouse mAb and P73 siRNA (h):(sc-36167) were purchased from Santa Cruz Biotechnology (Santa Cruz, CA). Control siRNA and YAP siRNA were synthesized by GenePharma Co, Ltd. (Shanghai, China). Alexa Fluor 568-labeled donkey anti-rabbit secondary antibody, Pro-Long Gold Antifade Mountant with DAPI and Lipofectamine RNA-iMAX were obtained from Invitrogen Life Science. The Annexin-V-FITC apoptosis kit was purchased from BD Biosciences.

### Cell viability assay

The Cell Counting Kit-8 (CCK-8; CK04, DOJINDO) was used to assess cell viability according to the manufacturer's protocol. Cells were seeded in 96-well plates and cultured for 24–48 h. When the monolayer of cells was 90% confluent, the HPMECs were exposed to various concentrations of LPS or CHX for 24 h. Next, 10 μl of CCK-8 solution was added to each well, and the cells were incubated for an additional 4 h. The absorbance at 450 nm was measured using a micro-plate reader. The cell survival rate was calculated using the average of pooled data from three separate experiments of six wells.

### Cell apoptosis analysis

HPMECs were treated separately with medium, LPS, CHX and both LPS and CHX for 24 h with or without pretreatment with special siRNA. The cells were then washed twice with pre-chilled PBS and re-suspended in 1 × binding buffer. HPMEC apoptosis was quantified by staining with 5 μl of Annexin V-fluorescein isothiocyanate (FITC) and 5 μl of PI. After incubation with Annexin V/PI for 10 min in the dark, 300 μl of 1 × binding buffer was added to the samples followed by flow cytometry analysis.

### Western blot analysis

Total protein was extracted from the HPMEC monolayers, and protein expression was analyzed by Western blotting. The protein samples from the HPMEC lysates were separated by 6–12% sodium dodecyl sulfate polyacrylamide gel electrophoresis (SDS-PAGE) according to the molecular weight of the target proteins and then electro-transferred to a PVDF membrane. The membranes were blocked with Tris-buffered saline (TBS) and Tween-20 containing 5% nonfat milk at room temperature for 1 h and then incubated with antibodies against the target proteins (diluted 1:1000) overnight at 4°C. The membranes were incubated with the appropriate HRP-linked secondary antibodies (1:2000) at room temperature for 1 h. The signal intensities were compensated using β-actin or glyceraldehyde 3-phosphate dehydrogenase (GAPDH) as internal controls. Finally, the bands were developed with Western blot luminal reagent (Millipore, Billerica, MA).

### Immunoprecipitation

To verify the complex formation between P-YAP (Y357) and P73 protein, HPMECs were lysed in 100 μl of RIPA+PMSF (1:100) containing protease inhibitor cocktail (1:100). The EC extracts were incubated overnight with P73 antibody (diluted 1:100), followed by incubation for 2 h with 30 μl of protein A agarose beads (Santa Cruz Biotechnology) according to the manufacturer's protocol. The precipitates were washed three times with lysis buffer on ice and then probed with anti-phospho-YAP (Y357) antibody (diluted 1:1000) by Western blot.

### Transfection with siRNA *In vitro*

YAP siRNA and P73 siRNA were transfected into HPMECs at an optimal concentration using Lipofectamine RNA-iMAX according to the manufacturer's instructions. Duplex siRNA was constructed against sequences encoding YAP positions (5′-CUG CCA CCA AGC UAG AUA ATT -3′ and 5′-UUA UCU AGC UUG GUG GCA GTT -3′). A scrambled negative control siRNA (5′-UUC UCC GAA CGU GUC ACG UTT-3′ and 5′-ACG UGA CAC GUU CGG AGA ATT-3′) was also included. P73 siRNA (h) is a target-specific 19–25-nt siRNA that is designed to knockdown P73 gene expression. The ability of the RNA interference molecules to knockdown target proteins expression was analyzed by Western blot analysis at 72 h after transfection. Transfection of the FITC-labeled nonspecific siRNA demonstrated that the transfection efficiency reached 85% in HPMECs.

### Immunofluorescent and immunocytochemical staining

For P-YAP (Y357) protein staining, HPMECs were grown to a density of 90% in Millicell EZ SLIDE 8-well glass plates (Millipore NO.PEZGS0816) and treated with LPS (1 μg/ml) and CHX (10 μg/ml) for 24 h. The monolayers were fixed with 4% paraformaldehyde for 30 min, washed, and blocked in phosphate-buffered saline containing 10% bovine serum albumin and 0.1% Triton X-100 for 30 min. The monolayers were incubated with anti-YAP (phospho-Y357) rabbit polyclonal antibody (1:200) overnight at 4°C, followed by Alexa Fluor 568-labeled donkey anti-rabbit secondary antibody (1:250) for 1 h. Before immunofluorescence analysis, anti-fade mountant containing DAPI was added to the samples, and the fluorescence images of P-YAP (Y357) mounted on glass coverslips were captured by microscopy (Nikon, Tokyo, Japan). Immunocytochemical staining of P-YAP (Y357) was visualized using the DAB system according to the manufacturer's instructions.

### Statistical analysis

All results are expressed as the means ± SEM of at least 3 independent experiments. Differences between groups were compared using the *t*-test and one-way ANOVA with Bonferoni post-hot analysis. Differences were considered significant at *P* < 0.05.

## Results

### CHX is required for LPS-induced HPMEC apoptosis *In vitro*

**Figure 1:**
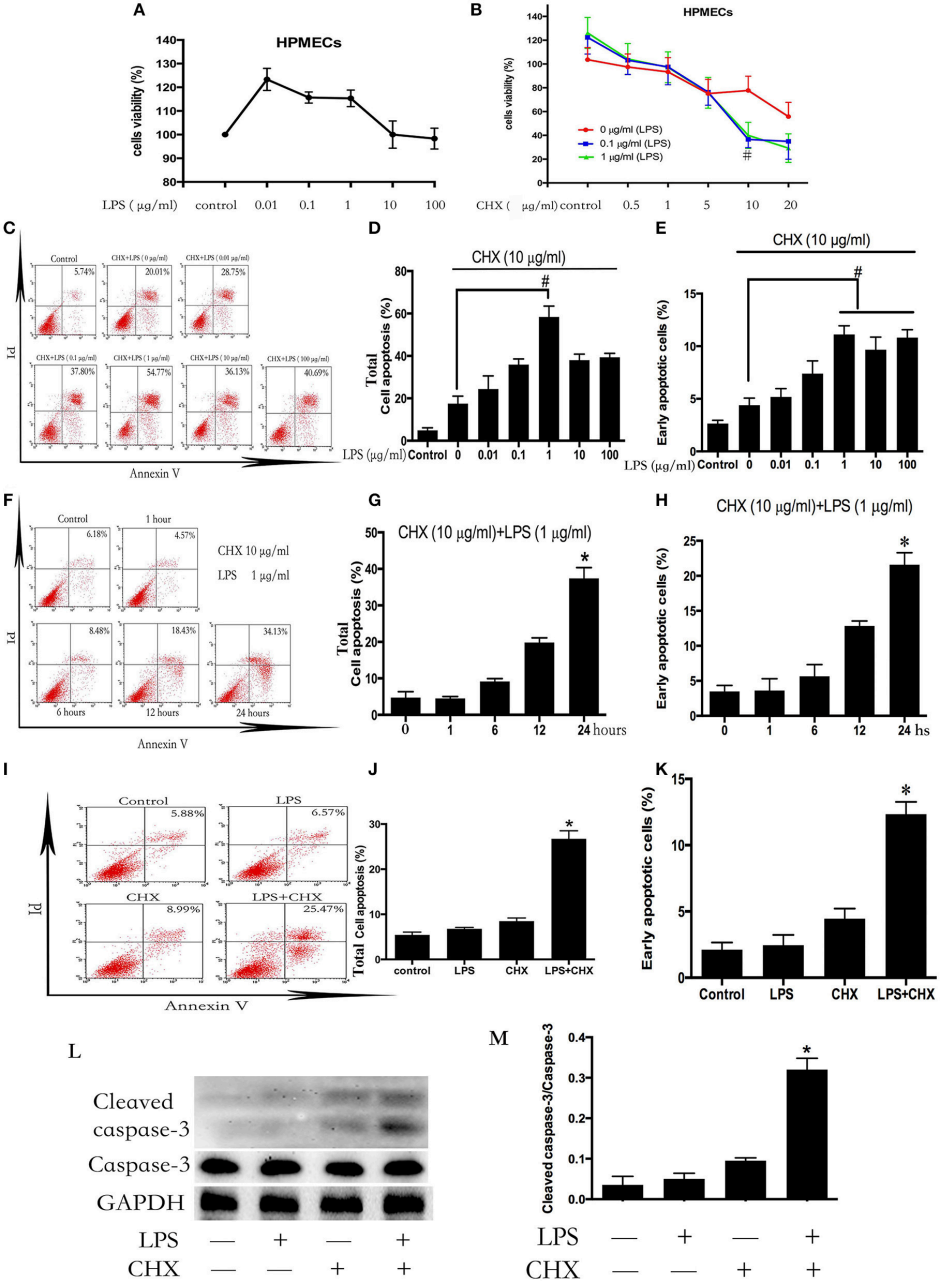
**LPS induces HPMEC apoptosis in the presence of CHX**. The viability of HPMECs was assessed after incubation with different concentrations of LPS (0, 0.01, 0.1, 1, 10, 100 μg/ml) for 24 h **(A)**. HPMECs were further incubated with different concentrations of LPS (0, 0.1, 1 μg/ml) and CHX (0–20 μg/ml) for 24 h, and viability was determined using the CCK-8 assay. When concentration of CHX was 10 μg/ml, the combination of LPS and CHX induced obvious cells death **(B)**. ^#^*P* < 0.05 vs. the comparator group without LPS treatment. HPMECs were exposed to CHX (10 μg/ml) and different concentration of LPS (0, 0.01, 0.1, 1, 10, 100 μg/ml) for 24 h, and apoptosis was measured by Annexin V/PI staining. The total apoptosis rate is expressed as the sum of the right upper and right lower quadrants. The early apoptosis rate is expressed as the right lower quadrants. The peak of apoptosis occurred at a LPS concentration of 1 μg/ml **(C)**. The results are presented as a histogram showing the percentage of total apoptosis cells and early apoptosis cells **(D,E)**. ^#^*P* < 0.05 vs. the comparator group treated only with CHX. HPMECs were incubated with CHX (10 μg/ml) and LPS (1 μg/ml) for the indicated times, and the apoptosis rate peaked at 24 h **(F)**. The results are presented as a histogram showing the percentage of total apoptosis cells and early apoptosis cells **(G,H)**. ^*^P < 0.05 vs. the control group. The combination of LPS (1 μg/ml) and CHX (10 μg/ml), but not LPS or CHX alone, induced obvious HPMEC apoptosis after 24 h flow cytometry **(I)**. The results are presented as a histogram showing the percentage of total apoptosis cells and early apoptosis cells **(J,K)**. ^*^P < 0.05 vs. the control group. Cleaved caspase-3 expression was detected in HPMECs incubated with LPS (1 μg/ml) and CHX (10 μg/ml) for 24 h **(L,M)**. Cells treated with medium alone were used as a negative control. ^*^P < 0.05 vs. the negative control group.

HPMECs were stimulated with different concentrations of LPS (0–100 μg/ml) for 24 h. The survival rate of the ECs was detected using CCK-8 (Figure 1A). Incubation with LPS alone did not induce HPMEC death. Subsequently, HPMECs were incubated with LPS and CHX simultaneously at various concentrations (LPS at 0–1 μg/ml and CHX at 0–20 μg/ml). After 24 h, the CCK-8 analysis revealed that the combination of LPS and CHX decreased the survival rate of HPMECs. In contrast to using CHX alone, the decrease in EC survival rate caused by the combination of LPS and CHX peaked at 10 μg/ml of CHX (Figure 1B). We further used an Annexin-V/PI apoptosis kit to quantify the numbers of apoptotic HPMECs. Both the total apoptosis rate and early apoptosis rate reached a maximum at 10 μg/ml of CHX and 1 μg/ml of LPS compared with the control group after 24 h (Figures 1C–H). The combination of LPS and CHX, but not LPS or CHX alone, induced HPMEC apoptosis (Figures 1I–K). These data were further confirmed by immunoblotting analysis of cleaved caspase-3 (Figures 1L,M).

### YAP is activated via phosphorylation of yap on Y357 during LPS-induced HPMEC apoptosis

**Figure 2:**
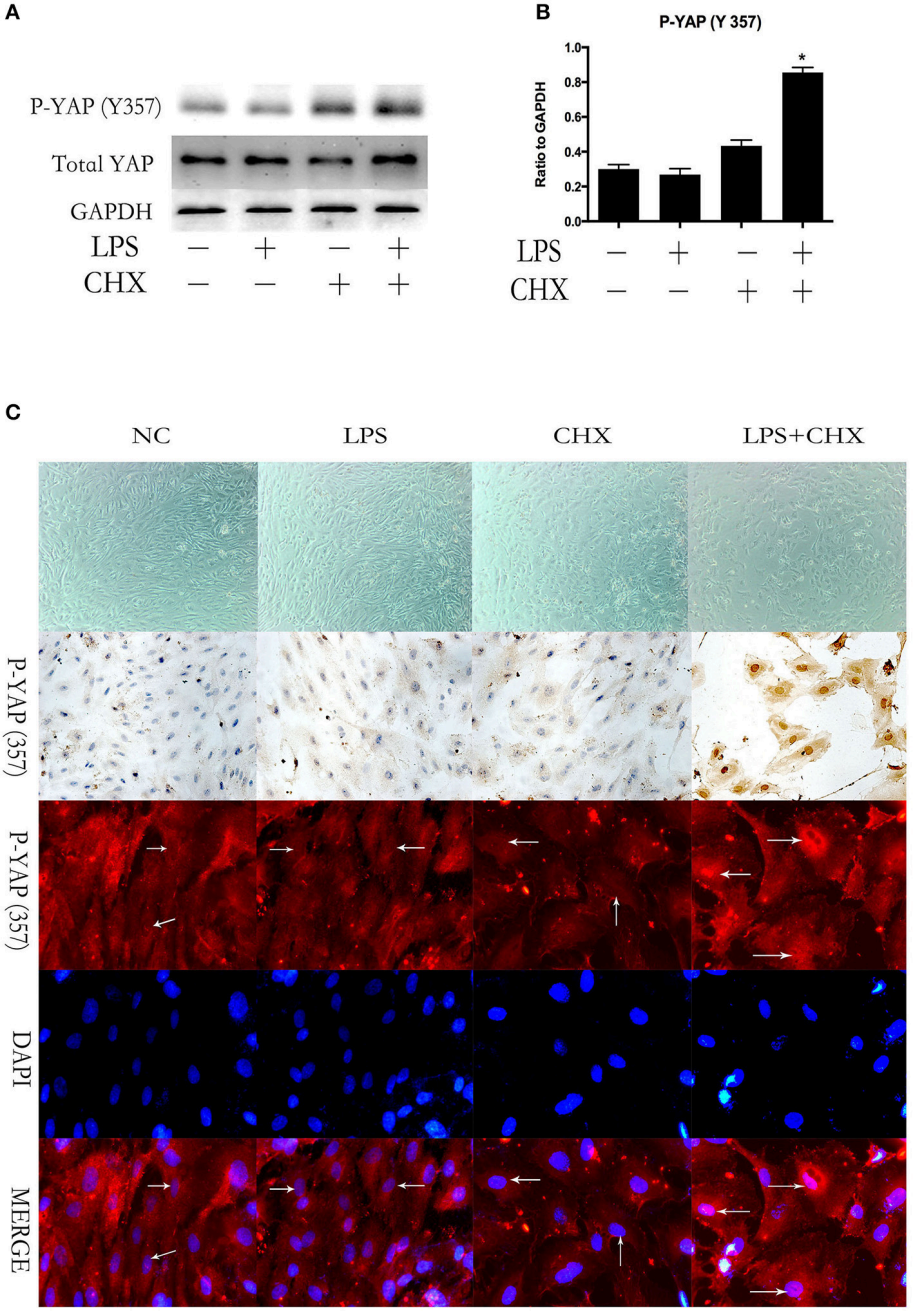
**YAP is activated during LPS-induced HPMEC apoptosis**. Expression of YAP and P-YAP (Y357) in HPMECs at the indicated time points after stimulation with LPS (1 μg/ml) and CHX (10 μg/ml) **(A,B)**. Cells treated with medium alone were used as the negative control. ^*^P < 0.05 vs. the negative control group. HPMECs were exposed to LPS (1 μg/ml) and CHX (10 μg/ml) before fixation and staining with anti-P-YAP (Y357) antibody as described in Materials and Methods **(C)**. P-YAP (Y357) (red) and nuclear staining with DAPI (blue) were visualized by immunofluorescence microscopy. Simultaneously, P-YAP (Y357) (brown) was detected by immunocytochemistry after incubation with LPS (1 μg/ml) and CHX (10 μg/ml). White arrows represent the expression of P-YAP (357) in the nucleus in HPMECs.

We further investigated the expression of YAP during LPS plus CHX-induced HPMEC apoptosis. After incubating HPMECs with LPS and CHX for 24 h, the expression of total YAP and the phosphorylation of YAP on Y357 were evaluated by Western blotting (Figure 2A). The data revealed no significant differences among the groups in total YAP expression. However, the combination of LPS and CHX significantly increased P-YAP (Y357) expression when compared with the medium, LPS or CHX groups alone (Figure 2B). These results were further confirmed by immunocytochemical and immunofluorescent staining for P-YAP (Y357) protein in HPMEC. We found that the phosphorylation of Y357 in YAP (colored red in the immunofluorescence images and brown in the immunocytochemistry images) primarily occurred and accumulated in HPMEC nuclei after stimulation with LPS and CHX (Figure 2C).

### Inhibition of YAP protein attenuated LPS-induced HPMEC apoptosis

**Figure 3:**
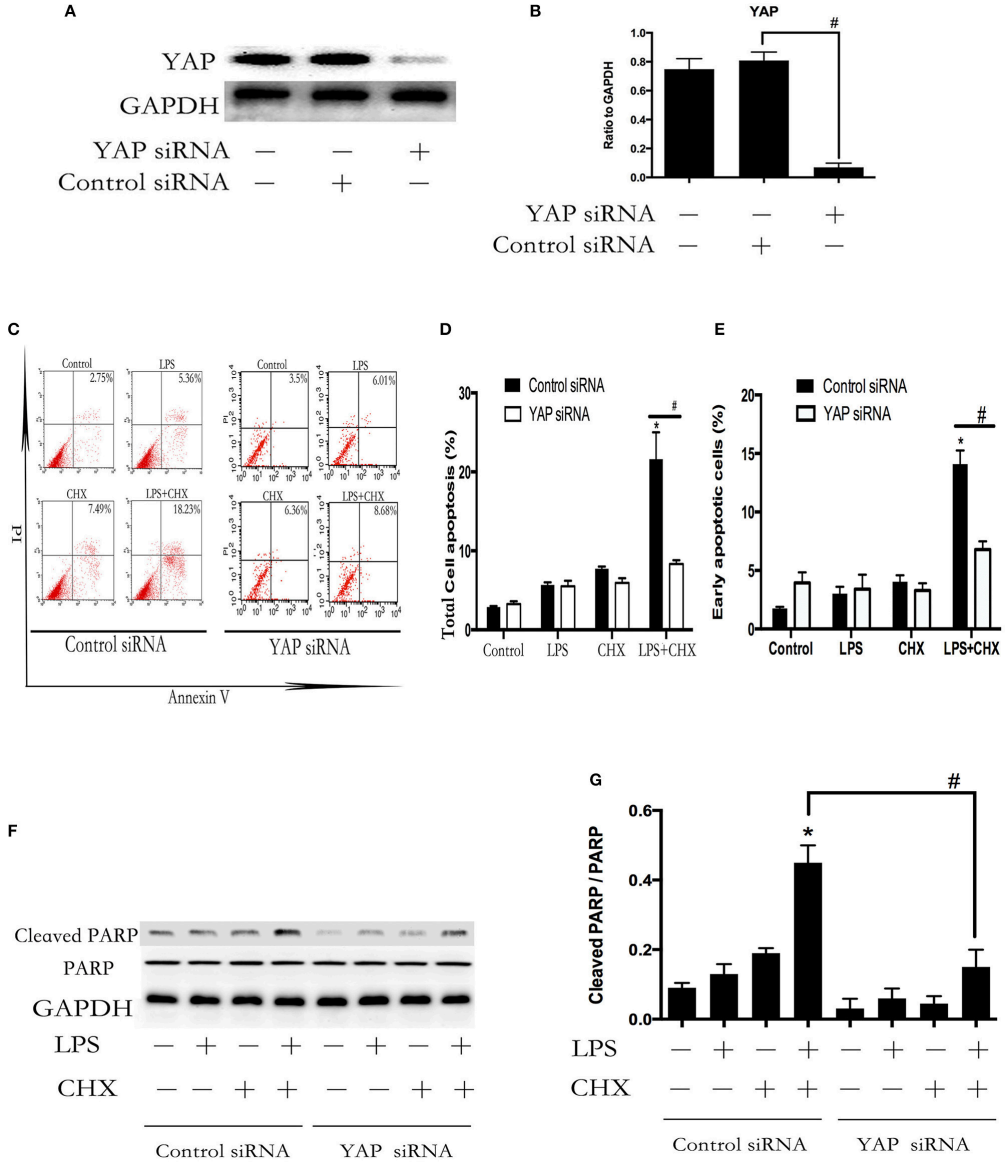
**YAP protein accelerates LPS-induced HPMEC apoptosis**. Western blot analysis of YAP depletion in HPMECs **(A,B)**. ^#^*P* < 0.05 vs. the control siRNA group. After transfection with YAP siRNA or control siRNA for 6 h, HPMECs were treated with LPS (1 μg/ml) and CHX (10 μg/ml) for the indicated times, and apoptotic cells was detected by Annexin V/PI staining **(C)**. The percentage of total apoptotic cells and early apoptotic cells are presented as a histogram showing results obtained by flow cytometry **(D,E)**. ^*^P < 0.05 vs. the control group. ^#^*P* < 0.05 vs. the corresponding LPS and CHX treatment group. HPMECs were transfected with YAP siRNA or control siRNA for 6 h and then stimulated with medium alone, LPS, CHX, or the combination of LPS and CHX for 24 h. The levels of cleaved PARP were determined by Western blot analysis **(F)**. The Western blotting results are presented as a histogram showing the band intensity values **(G)**. Cells treated with medium alone were used as the negative control. ^*^P < 0.05 vs. the negative control group. ^#^*P* < 0.05 vs. the corresponding LPS and CHX treatment groups.

To investigate whether YAP was involved in the combined LPS and CHX-induced EC apoptosis, we examined HPMECs transfected with YAP siRNA. The effect of YAP knockdown was confirmed by Western blot analysis (Figures 3A,B). Flow cytometry demonstrated that combined LPS and CHX-induced HPMEC apoptosis was blocked by YAP siRNA at the indicated times (Figures 3C–E). PARP is associated with DNA repair and is a significant marker of apoptosis (Jemal et al., 2011). The above result was further confirmed by Western blot analysis of cleaved PARP expression (Figures 3F,G).

### YAP interacts with transcription factor P73 during LPS-induced HPMEC apoptosis

**Figure 4:**
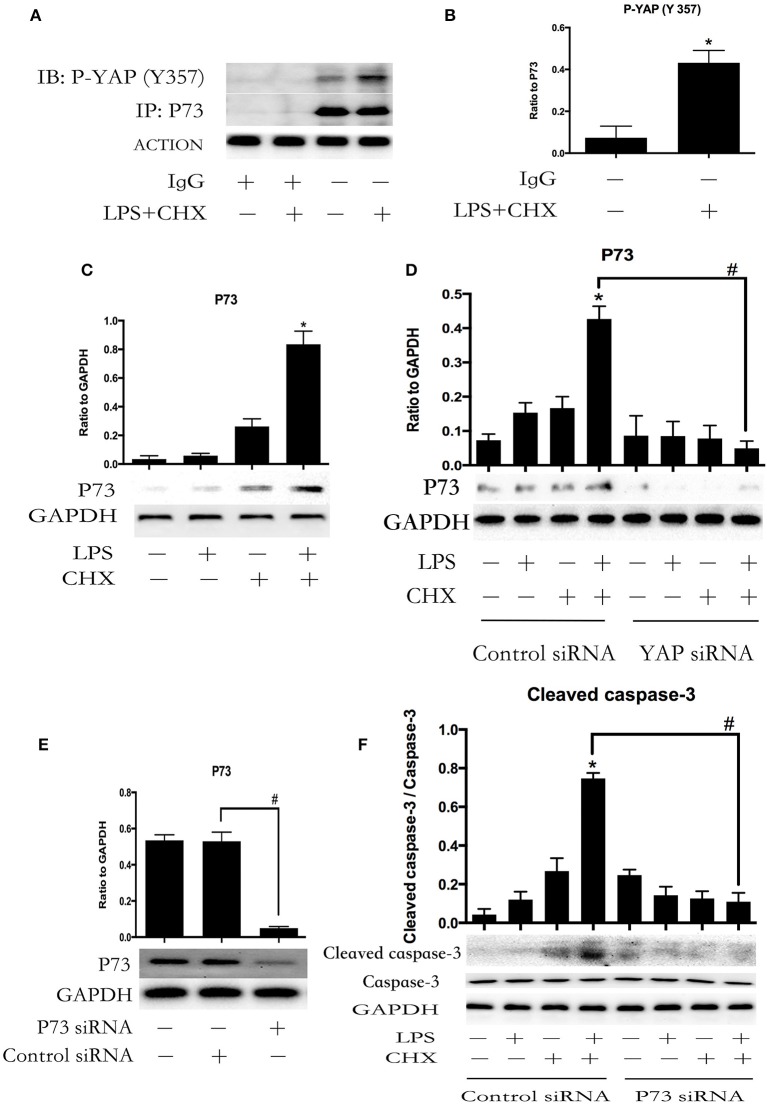
**YAP interacts with P73 during LPS-induced HPMEC apoptosis**. P73 and phosphor-YAP (Y357) formed the signaling complex. Co-IP was performed to detect the P73-YAP complex at the indicated times after stimulation with LPS and CHX. Western blotting was performed to detect P-YAP (Y357) in the IP complexes **(A)**. The expression of phosphor-YAP (Y357) in IP complexes is presented as a histogram showing the band intensity values **(B)**. ^*^P < 0.05 vs. the untreated group. HPMECs were incubated with LPS (1 μg/ml) and CHX (10 μg/ml) for 24 h, and P73 protein was detected by Western blot analysis **(C)**. ^*^P < 0.05 vs. the control group. HPMECs were transferred with YAP siRNA or control siRNA for 6 h and then stimulated with medium alone, LPS, CHX, or the combination of LPS and CHX for 24 h. The levels of P73 were determined by Western blot analysis **(D)**. Cells treated with medium alone were used as the negative control. ^*^P < 0.05 vs. the negative control group. ^#^*P* < 0.05 vs. the corresponding LPS and CHX treatment group. Western blot analysis of P73 depletion in HPMECs **(E)**. ^#^*P* < 0.05 vs. the control siRNA group. After transfection with P73 siRNA or control siRNA for 6 h, HPMECs were treated with LPS (1 μg/ml) and CHX (10 μg/ml) for the indicated times, and cleaved caspase-3 was detected by Western blot analysis **(F)**. Cells treated with medium alone were used as the negative control. ^*^P < 0.05 vs. the negative control group. ^#^*P* < 0.05 vs. the corresponding LPS and CHX treatment group.

To confirm whether YAP protein was involved in LPS plus CHX-induced HPMEC apoptosis via the interaction with P73, co-immunoprecipitation was used to verify the relationship between P-YAP (Y357) and P73. The stimulation of LPS and CHX led to the formation of YAP/P73 transcriptional complex in HPMECs, as determined by immunoblotting with antibodies against P-YAP (Y357) after immunoprecipitation of P73 (Figures 4A,B). Meanwhile, Immunoblotting analysis revealed that combination of LPS and CHX significantly induced P73 expression (Figure 4C). After transfecting with YAP or control siRNA for 6 h, the HPMECs were treated with medium, LPS, CHX or the combination of LPS and CHX for 24 h. We further found that YAP knockdown effectively down-regulated the expression of P73 in HPMECs (Figure 4D). We examined HPMECs transfected with P73 siRNA. The effect of P73 knockdown was confirmed by Western blot analysis (Figure 4E). The results showed that P73 depletion inhibited LPS plus CHX-induced caspase-3 activation in HPMECs (Figure 4F)

### YAP/P73 signaling is implicated in LPS-induced HPMEC apoptosis via regulation of BAX and BCL-2 expression

**Figure 5:**
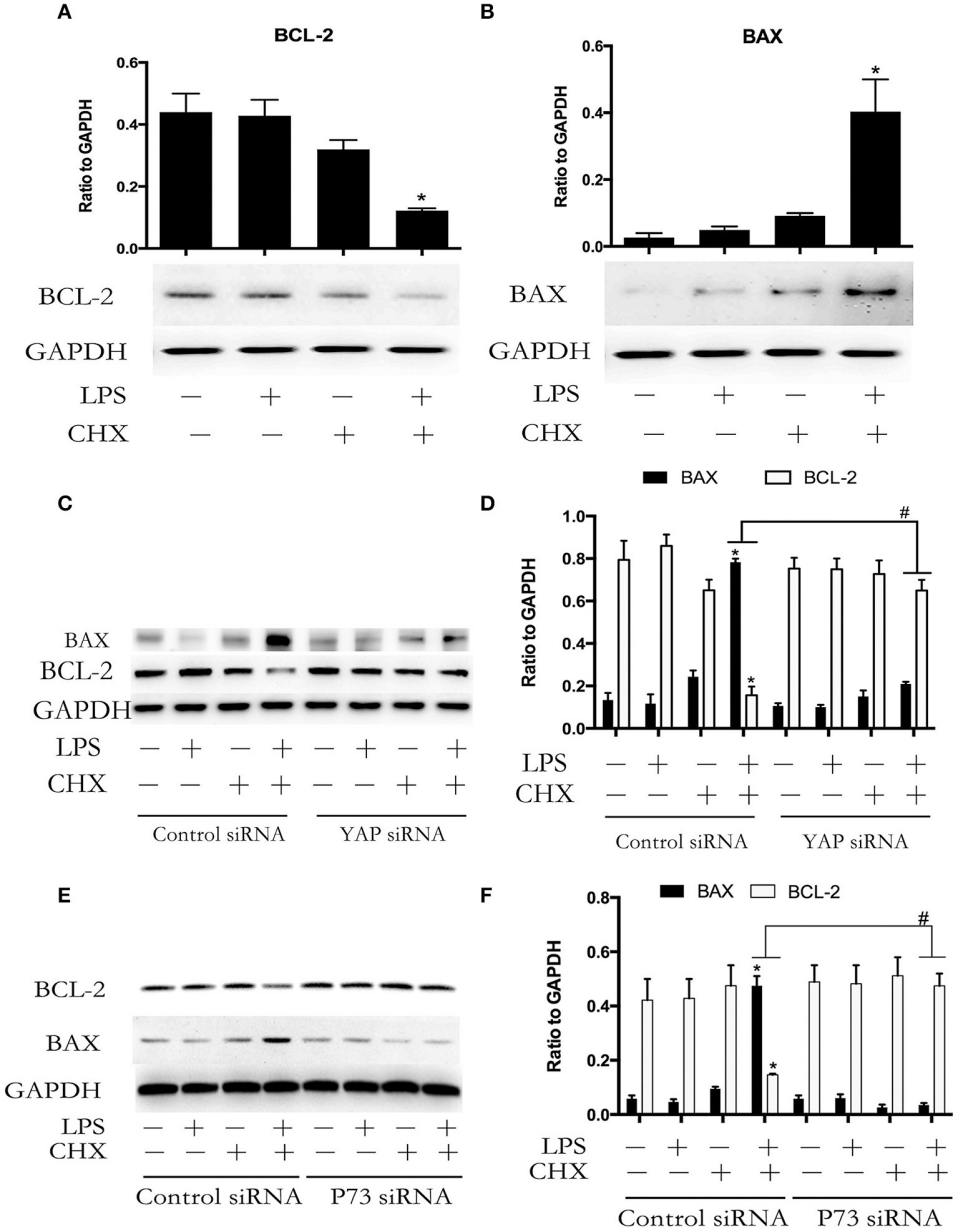
**YAP/P73 signaling is involved in LPS-induced HPMEC apoptosis via regulation of BAX and BCL-2**. The HPMECs were exposed to the combination of LPS (1 μg/ml) and CHX (10 μg/ml) for 24 h, and BAX and BCL-2 expression levels were measured by Western blot analysis **(A,B)**. After transfection with YAP siRNA or control siRNA, HPMECs were treated with LPS (1 μg/ml) and CHX (10 μg/ml) for the indicated times, and BAX and BCL-2 were detected by Western blot analysis **(C)**. The Western blotting results are presented as a histogram showing the band intensity values **(D)**. Cells treated with medium alone were used as the negative control. ^*^P < 0.05 vs. the negative control group. ^#^*P* < 0.05 vs. the corresponding LPS and CHX treatment group. HPMECs were transfected with P73 siRNA or control siRNA for 6 h and were then stimulated with medium alone, LPS, CHX, or the combination of LPS and CHX for 24 h. The levels of BAX and BCL-2 protein were determined by Western blot analysis **(E)**. The Western blotting results are presented as a histogram showing the band intensity values **(F)**. Cells treated with medium alone were used as the negative control. ^*^P < 0.05 vs. the negative control group. ^#^*P* < 0.05 vs. the corresponding LPS and CHX treatment groups.

BCL-2 family proteins-mediated intrinsic apoptosis pathway plays an important role in LPS-induced EC apoptosis (Wang et al., 2007). We further determined the expression of the pro-apoptotic protein BAX and the anti-apoptotic protein BCL-2 during LPS plus CHX-induced HPMEC apoptosis. Western blot analyses revealed that the combination of LPS and CHX promoted BAX expression and inhibited BCL-2 expression in HPMEC at the indicated times (Figures 5A,B). To determine the role of YAP/P73 signaling on the expression of BAX and BCL-2 during HPMEC apoptosis, HPMECs were transfected with YAP siRNA or P73 siRNA. The results showed that both YAP and P73 depletion reversed LPS and CHX-induced up-regulation of BAX and down-regulation of BCL-2 (Figures 5C–F).

## Discussion

The hyper-permeability of the microvasculature complicates sepsis (Gustot, 2011). Human endothelial cells undergo injury and apoptosis resulting in detachment from the vessel wall in patients with sepsis (Schlichting et al., 2011; Zahran et al., 2016). Lung micro-vascular EC apoptosis-mediated vascular hyper-permeability acts as an important initiator of ALI in response to LPS (Damarla et al., 2014). However, the mechanism underlying LPS-induced HPMEC apoptosis *in vitro* is not well understood. In the present study, We showed that YAP accelerated LPS-induced HPMEC apoptosis through its phosphorylation activation and accumulation in EC nucleus. The localization of YAP in the nucleus further promoted the combination between YAP and its target transcription factor P73 to regulate the expression of BCL-2 and BAX. Our results found that YAP, P73, BAX, BCL-2 and caspase-3 were implicated in HPMEC apoptosis triggered by LPS plus CHX. These findings showed that the YAP/P73/(BAX and BCL-2)/caspase-3 signaling pathway was likely vital for mediating the development of lung injury under sepsis.

Previous reports demonstrated that LPS alone could rapidly induce apoptosis of human ECs *in vitro* (Kim et al., 2002; Damarla et al., 2014). However, our study found that stimulating HPMECs with LPS did not lead to cellular death. Instead, LPS induced slight cell proliferation, potentially because human endothelial cells incubated with LPS activate both pro-apoptotic and also anti-apoptotic pathways *in vitro* (Bannerman et al., 2001, 2004).

FLICE-like inhibitor protein (FLIP) was known as the core anti-apoptotic protein induced by LPS in human ECs. Shiga-like toxin (SLT) and Shiga toxin, which are produced by *Shigella dysenteriae* serotype 1 and certain strains of *Escherichia coli*, have been clearly established to inhibit expression of FLIP and sensitize ECs to LPS-induced apoptosis (Louise and Obrig, 1992; Erwert et al., 2002). However, in the parallel studies between micro-vascular ECs and macro-vascular ECs, the combination of LPS and SLT only induced cytotoxicity in the macro-vascular-derived human umbilical vein ECs, not in the micro-vascular ECs (Obrig et al., 1993). Cycloheximide (CHX) is a nonspecific inhibitor of protein synthesis. It also sensitizes human ECs to LPS-induced apoptosis by both the rapid degradation of preexisting FLIP and the inhibition of de novo synthesis of FLIP molecules (Bannerman and Goldblum, 2003). Many previous studies have reported that the micro-vascular ECs apoptosis model *in vitro* mainly induced by the stimulation of LPS plus CHX (Choi et al., 1998; Wang et al., 2007; Karahashi et al., 2009). In the study, we further explored the mechanism of LPS-induced human pulmonary micro-vascular ECs in the presence of CHX.

Some previous studies have observed that CHX (40 μg/ml) can sensitize LPS-induced human ECs apoptosis *in vitro* (Bannerman et al., 2004; Karahashi et al., 2009). Consistent with the previous study, we also found that LPS induced a remarkably decreased rate of survival and increased rate of apoptosis in HPMECs in the presence of lower concentration of CHX (10 μg/ml). In order to find out the optimal concentration of LPS in the study of HPMECs apoptosis *in vitro*, we further used different concentrations of LPS (0, 0.01, 0.1, 1, 10, and 100 μg/ml) to incubate primary HPMECs under the presence of CHX (10 μg/ml). We found that the peak of total apoptosis rate and early apoptosis rate of HPMECs appeared at concentrations of 1 μg/ml. Though these observations are analogous to previous reports in which authors showed that LPS (0.1 μg/ml) plus CHX (50 μg/ml) activated a FADD-dependent apoptotic pathway in the human dermal micro-vascular cell line (HMEC-1) (Choi et al., 1998), the optimal concentration of LPS (1 μg/ml) in our study is 10 times higher than the previous reports. The reasons may be that the primary HPMECs is more sensitive to the stimulation of CHX and need higher concentration of LPS when the stimulating concentration of CHX (10 μg/ml) is low. In addition, because primary HPMECs are sensitive to the lack of endothelial cell growth supplement and fetal bovine serum in the endothelial cell medium, we used complete medium plus LPS and/or CHX to study HPMECs apoptosis *in vitro*. The anti-apoptosis roles of endothelial cell growth supplement and fetal bovine serum may counteract the pro-apoptosis roles of LPS in the physiological concentration. Therefore, it may be the reasons that HPMECs apoptosis is induced with high concentration of LPS (1 μg/ml) in our study.

YAP is a crucial transcriptional co-activator shuttling between nucleus and cytoplasm and its activity is tightly regulated by several upstream kinases (Yagi et al., 1999). YAP phosphorylation on serine 127 by Akt increases the interaction of YAP with the cytosolic 14-3-3 protein leading to inactivation of YAP and subsequent YAP degradation in cytoplasm (Basu et al., 2003). On the contrary, c-Abl phosphorylates YAP on tyrosine 357 results in activation of YAP, which is implicated in promoting cancer cells apoptosis (Levy et al., 2008; Jie et al., 2013). In our study, we demonstrated for the first time that YAP protein was activated in HPMECs during LPS and CHX-induced HPMEC apoptosis. In addition, we also showed that the activated YAP accumulated in HPMEC nucleus after stimulation with LPS and CHX. Importantly, we found that knockdown of YAP protein attenuated the apoptosis induced by LPS plus CHX. Thus, above results revealed that YAP protein was required for LPS and CHX-induced HPMEC apoptosis and that apoptosis was associated with nuclear accumulation of activated YAP.

As a transcriptional co-activator, YAP protein needs to bind target transcription factors to stimulate downstream gene expression (Li et al., 2010). Previous studies have showed that YAP protein positively regulates transcription factor P73 and Egr-1 in promoting cellular apoptosis through mediating the expression of death-associated genes (Lapi et al., 2008; Zagurovskaya et al., 2009). Interestingly, YAP activation by phosphorylation on T357 displays higher affinity to P73 (Levy et al., 2008). Our study found that the combination of LPS and CHX induced augment and nuclear localization of P-YAP (T357) in HPMECs. However, whether P-YAP (T357) interacts with P73 during LPS plus CHX-induced EC apoptosis is unclear. Here, we showed that the interaction between P-YAP (T357) and P73 increased in HPMECs after treatment of LPS and CHX. This result supported the idea that YAP protein may be involved in LPS plus CHX-induced HPMEC apoptosis via the formation of YAP/P73 transcriptional complex. We also found that stimulation of LPS and CHX promoted the augment of P73 protein. In addition, knockdown of YAP effectively inhibited LPS plus CHX-induced P73 expression. These results may be due to the reason that YAP competes with ITCH to bind P73, which inhibits degradation of P73 and selectively co-activates P73 (Levy et al., 2007). Moreover, knockdown of P73 also inhibited the LPS plus CHX-induced caspase-3 activation in HPMECs. Therefore, our results indicated that the formation of YAP/P73 transcriptional complex was crucial for mediating LPS plus CHX-induced HPMEC apoptosis.

BAX and BCL-2 are the transcriptional target proteins of P73 (Tiwary et al., 2011). BAX and BCL-2 activate caspases by promoting the release of cytochrome C from mitochondrion (Adrain and Martin, 2001). Studies have proved that the BAX expression of lung is increased and BCL-2 expression of lung is decreased in sepsis-induced ALI model in mice (Chopra et al., 2009). Analogous to previous reports, we also observed BAX up-regulation and BCL-2 down-regulation in LPS plus CHX-incubated HPMECs, which coincided with the augment in YAP phosphorylation and P73 activation during LPS plus CHX-induced HPMEC apoptosis. However, whether YAP/P73 signaling pathway mediates the regulation of BAX and BCL-2 expression in LPS plus CHX-induced HPMEC apoptosis is still unknown. In the present study, inhibition of YAP/P73 signaling by siRNA reversed LPS plus CHX-induced up-regulation of BAX and down-regulation of BCL-2. The above observations are consistent with previous reports in which authors demonstrated that YAP or P73 respectively contributed to regulate the expression of BAX and BCL-2 in the process of different cancer cell apoptosis (Melino et al., 2004; Chipuk and Green, 2009; Xu et al., 2015). Taken together, our data suggested that YAP protein accelerated LPS plus CHX-induced HPMEC apoptosis via interaction with transcription factor P73, leading to caspase-3 activation through the up-regulation of BAX expression and down-regulation of BCL-2 expression. However, although CHX have been used as tool to promote sensitization to LPS-induced HPMECs apoptosis *in vitro*, it is unlikely that LPS and CHX co-exist in human body. So, the LPS plus CHX-induced HPMECs apoptosis model *in vivo* is constrained. Interestingly, SLT is a more clinically relevant type of protein synthesis inhibitor induced by bacteria, and micro-vascular ECs are a principal target of SLT-1 *in vivo* (O'Loughlin and Robins-Browne, 2001). *Escherichia coli* produce SLT-1 in addition to LPS. In this regard, whether YAP signaling involved in LPS plus SLT-1-induced HPMECs apoptosis and sepsis-associated ALI *in vivo* need to be studied further.

In conclusion, we demonstrated that YAP mediated LPS-induced HPMEC apoptosis *in vitro* via its phosphorylation activation and accumulating in nucleus to bind its target transcription factor P73 to stimulate BAX expression and inhibit BCL-2 expression. Our results raised the possibility that YAP/P73/(BAX and BCL-2)/caspase-3 pathway played an important role in regulating HPMEC apoptosis in sepsis-induced ALI.

## Author contributions

LY and XH made contributions to analysis and interpretation of data, drafting the article and revising it, and final approval of the version to be published; FG, ZZ, MC, and JT made contributions to acquisition of data, editing the manuscript; JH made substantial contributions to conception and design of the work and analysis and interpretation of data, drafting and revising the manuscript critically, and final approval of the version to be published.

### Conflict of interest statement

The authors declare that the research was conducted in the absence of any commercial or financial relationships that could be construed as a potential conflict of interest.
